# Association between Creativity and Memory with Cardiorespiratory Fitness and Lifestyle among Chilean Schoolchildren

**DOI:** 10.3390/nu13061799

**Published:** 2021-05-25

**Authors:** Felipe Caamaño-Navarrete, Pedro Á. Latorre-Román, Juan A. Párraga-Montilla, Cristian Álvarez, Pedro Delgado-Floody

**Affiliations:** 1 Faculty of Education, Universidad Católica de Temuco, Temuco 478000, Chile; marfel77@gmail.com; 2 Department of Didactics of Music, Plastic and Corporal Expression, University of Jaén, 23071 Jaén, Spain; platorre@ujaen.es (P.Á.L.-R.); jparraga@ujaen.es (J.A.P.-M.); 3 Quality of Life and Wellness Research Group API4, Laboratory of Human Performance, Department of Physical Activity Sciences, Universidad de Los Lagos, Osorno 5290000, Chile; cristian.alvarez@ulagos.cl; 4 Department of Physical Education, Sport and Recreation, Universidad de La Frontera, Temuco 478000, Chile

**Keywords:** creativity, memory, cardiorespiratory fitness, mediterranean diet, schoolchildren

## Abstract

The objective was to investigate the association between creativity and memory with cardiorespiratory fitness (CRF; i.e., CFR classification and $\dot{V}$O_2max_); lifestyle parameters (i.e., physical activity (PA), sleep duration, screen time (ST), and food habits); and anthropometric measures (i.e., body mass index (BMI), waist circumference (WC)) among Chilean schoolchildren. A total of 248 schoolchildren (137 boys, 111 girls, 11.80 ± 1.17 and 11.58 ± 1.09 years, respectively) participated in the cross-sectional study. Creativity, memory, concentration, and selective attention and lifestyle (PA, ST, sleep duration, and Mediterranean diet (MD) adherence) were measured using a standard questionnaire. CRF (measured by the 20 m shuttle run test and expressed as maximum oxygen consumption $(\dot{V}$O_2max_) and anthropometric measures (BMI and WC) were also included. Creativity showed a positive association with $\dot{V}$O_2max_ (mL/kg/min) (β; 0.209, 95% CI; 0.02–0.40, *p* = *p* < 0.05) and MD Adherence (score) (β; 0.206, 95% CI; 0.01; 0.74, *p* = *p* < 0.05). Long-term memory reported a positive association with CRF (β; 1.076, 95% CI; 0.02–2.13, *p* = *p* < 0.05). An increase in CRF levels, together with healthy food habits and normal nutritional status, should be a target for community- and school-based interventions to promote cognitive development in creativity and memory among schoolchildren.

## 1. Introduction

Childhood and adolescence are sensitive periods for cognitive development [1,2]. Executive function is the meta-cognitive function necessary for conducting complex and goal-oriented operations [3]. Executive function plays an important role in children’s and adolescents’ academic performance [4]. The results of a study by Demetriou et al. [5] indicated that cognitive ability was the strongest predictor of school performance in childhood and adolescence. Similarly, it has been found that different cognitive components related to executive function are associated with academic skills, including creativity and memory [6]. Creativity is an important human activity and is considered a key element to cognition, and among the least investigated [7]. Creativity has also shown a positive association with academic achievement [8], and many authorities have begun to adopt educational policies designed to promote creativity [9]. Memory is not a singular construct, and the most commonly studied types of memory are short-term memory, working memory, and long-term memory [10]. Different cognitive components (i.e., types of memory) are associated with academic skills [6], and attention capacity is a crucial element for comprehension and learning processes [11].

Assessment of physical fitness among schoolchildren should be essential for controlling the health of this population. Adequate cardiorespiratory fitness (CRF) in childhood may be important to the development of cognitive processes [12]. CRF is a direct indicator of an individual’s cardiovascular and respiratory systems’ overall capacity to perform physical activities [3]. In recent years, the study of cognitive processes has increased in the field of physical activity (PA) [13]. Current evidence suggests that better levels of CRF have positive effects on children’s cognition [14]. Aditionally, among children and youth, previous studies have shown that individuals with high levels of CRF had better performances in physical education [3]. Such studies have been consistent in reporting a positive association between CRF and academic performance [3,15]. Regarding creativity, the results of the study by Latorre et al. [12] conducted in children showed that CRF was a predictor for creativity. Likewise, better CRF was also associated with better memory (i.e., working memory) [16]. A positive relationship between CRF, selective attention and concentration has also been reported [17]. Several mechanisms have been proposed to explain the association of CRF with cognition [18]. For example, subjects who demonstrated better CRF functions (as measured by V˙V˙O_2*max*_) show faster reaction times and greater cerebral oxygenation, and cognitive processing is critically dependent on adequate blood flow to respond the energy and oxygen needs of the tissue [19]. Cognitive performance is tightly associated with CRF through cerebrovascular endothelial function and angiogenesis [20]. CRF may benefit brain health and plasticity, possibly via a brain-derived neurotropic factor (BDNF)-regulated mechanism [21].

Children’s lifestyle (i.e., diet, PA, etc.) has commonly been studied in the context of health [22]. However, it is reasonable to expect that lifestyle factors are intertwined with cognition and learning processes in children [23]. For example, Kim et al. reported a positive association between school performance and dietary habits [24]. Healthy diet habits (i.e., breakfast consumption), for example, may improve cognitive function and test grades [25]. However, little is known regarding the associations of nutrition with components of executive function in adolescence [26], especially among Chilean schoolchildren. It is important to consider that a Chilean national study of physical education focused on physical condition and anthropometric parameters showed that 70% of students need to improve their CRF [27]. Additionally, in Chile, a high prevalence of children with overweight and obesity have been reported (approximately 44% of students at the age of 13 years old) [28]. Therefore, it is considered a public health problem [22]. In this line, it is important to evaluate the association between CFR and nutritional status with executive function, particularly in Chilean schoolchildren. Likewise, to the best of our knowledge, no other study has explored the association of creativity and memory with CRF, lifestyle, and anthropometric parameters in Chilean schoolchildren. The objective of the present study was to determine the association between creativity and memory with CRF (i.e., CFR classification and $\dot{V}$O_2max_), lifestyle parameters (i.e., PA, screen time, sleep duration, and food habits), and anthropometric measures (i.e., body mass index and waist circumference) among Chilean schoolchildren. A secondary aim was to compare creativity, memory, concentration, and selective attention according to CRF levels (i.e., high and low) and nutritional status. 

## 2. Materials and Methods

### 2.1. Participants

A descriptive study with a cross-sectional design was developed. A total of 248 schoolchildren (137 boys and 111 girls, 11.80 ± 1.17 and 11.58 ± 1.09 years, respectively) from a subsidized private school, which are financed by a mixture of funding from central government and private contributions [29] in Temuco, Chile participated in the study.

The students who attended the school were of the same sociocultural level and lived in the same geographical area (urban). There were 33 subjects excluded (girls not meeting inclusion criteria or other reasons (*n* = 23); boys not meeting inclusion criteria or other reasons (*n* = 10)). The sample was intentional and non-probabilistic. The inclusion criteria were: Chilean schoolchildren aged between 10 and 14, without musculoskeletal diseases or physical/medical conditions that might alter the participants’ health and physical fitness levels and also children who complete every test administered as part of this study. Schoolchildren with intellectual or physical disabilities were excluded. The investigation complied with the Declaration of Helsinki (2013) and was approved by an Ethics Committee (ABR.19/8.TES Act). This study is part of a Doctoral Thesis. 

**Figure 1:**
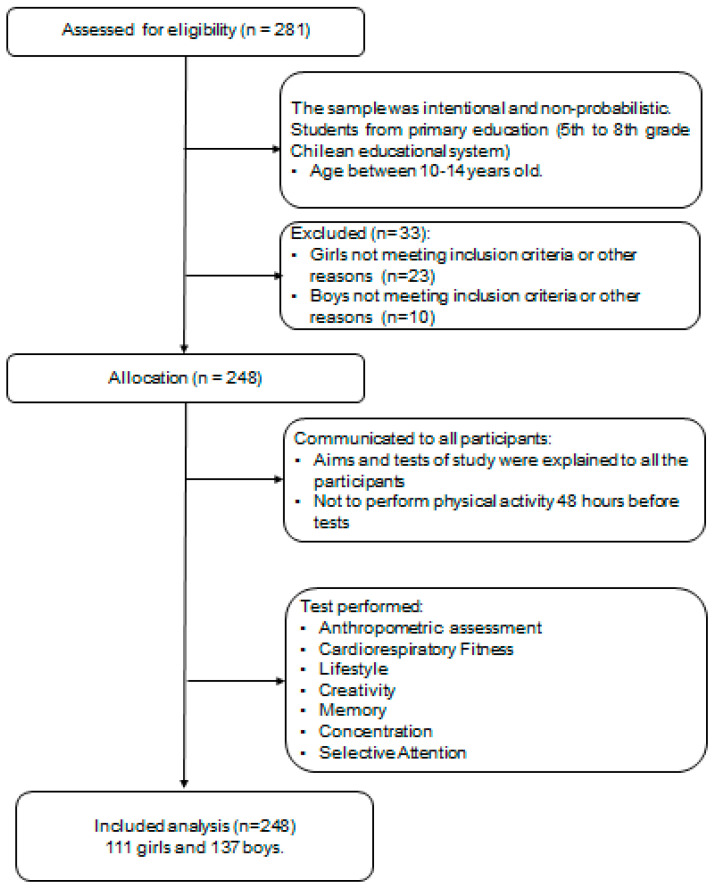
Study design.

Parents and guardians were informed about the study and provided written signed consent for participation. Additionally, all participants gave their written consent on the day of the assessment. Figure 1 shows the study design. 

### 2.2. Measures

#### 2.2.1. Creativity 

Creativity was measured using a CREA test [30] which provides a global quantitative measure of creativity [31]. The test is a timed 4-min divergent thinking test that contains a picture and asks respondents to generate questions about the picture [32]. From an image, the schoolchildren elaborated as many questions as possible for 4 min. A score was assigned to each question according to its quality and complexity based on criteria established by the authors in the manual for the test (1 = low, 2 = medium, 3 = high). In this study, we used the original version in Spanish. The test manual reports strong reliability, convergent validity with Guilford’s divergent thinking tasks [33], and has high and stable correlations over time with other instruments and measures of creativity [32]. 

#### 2.2.2. Memory

Memory capacity was measured by Ray’s Auditory Verbal Learning Test (RAVLT). A list of 15 words (list A) was presented in five consecutive trials, assessing (after each trial) the number of words remembered by the participant. They were immediately shown an interference list (list B), which they were asked to recall from memory. Then, the free memory of the first list of words was requested, and the process was repeated after 20 min [34]. A long-term memory test (after 20 min, at the end of the test battery, participants were asked to recall as many words as possible and to recognize the words within a list of 30 words) was used as an outcome measure [35]. A previous study showed that RAVLT showed reasonable test-retest reliability [36]. 

#### 2.2.3. Concentration and Selective Attention 

Concentration and attention capacity were measured with the d2 Test of Attention (d2), which has reliability from 0.95 to 0.98 [37]. The d2 consists of a paper and pencil test comprising 14 rows, each with 47 randomly interspersed ‘p’ and ‘d’ characters. Each character appears with 1 or 2 dashes placed above and/or below it [11]. The test takes 4 min and 30 s to be performed (20 s per line). Concentration was evaluated as: Number of hits—number of mistakes. Likewise, selective attention capacity was calculated as the number of processed elements—(omissions + mistakes) [38]. 

#### 2.2.4. Cardiorespiratory Fitness 

CRF was evaluated by the progressive Léger test. The students run between two parallel lines 20 m away from each other [39]. The last progression executed was recorded and calculated as the $\dot{V}$O_2max_ (mL/kg/min) using the Equation as follows: $\dot{V}$O_2_peak = 31.025 + 3.238 (V) − 3.248 (A) + 0.1536 (VA), where V is the velocity in km/h reached at the last stage and A stands for the student’s age [39]. In addition, students were divided into two groups: High/low CRF (i.e., $\dot{V}$O_2_peak outcome) according to previously cut-off points in school population as follows: 42 mL·kg^−1^·min^−1^ in boys and 35 mL·kg^−1^·min^−1^ in girls [40]. A higher $\dot{V}$O_2_peak indicated better CRF.

#### 2.2.5. Mediterranean Diet Adherence

The food habits of schoolchildren were determined by the Krece Plus test which is based on adherence to the Mediterranean diet (MD). The items have a score of +1 or −1 according to the established guidelines. The score from the Krece Plus test was categorized as follows: (1) >8, optimal MD; (2) 4–7, moderate MD adherence; and (3) ≤3, very low diet quality [41]. Higher scores indicate better food habits. 

#### 2.2.6. Levels of Physical Activity 

A Physical Activity Questionnaire (PAQ-C) was used to measure the PA levels of children. This instrument collects information about schoolchildren’s PA during the past 7 days [42]. Each item has a score between 1 and 5 (i.e., higher score means higher levels of PA). This self-administered instrument reported good reliability [42]. The results for PA are registered and quantified in hours per week. 

#### 2.2.7. Screen Time

The Krece Plus test was used to evaluated screen time [43]. This test is a quick questionnaire that classifies lifestyle based on the average number of hours spent watching television or playing video games daily. 

#### 2.2.8. Sleep Duration 

To evaluate children’s sleep duration, parents completed the Pediatric Sleep Questionnaire [44]. Parents or guardians answered questions referring to the quality and quantity of their children’s sleep. Correspondingly, this questionnaire has been reported with good reliability [45]. 

#### 2.2.9. Anthropometric Assessment 

A TANITA scale (model UM–028, Tokyo) was used to evaluate the children’s weight (kg). Children’s height (m) was measured with a Seca^®^ stadiometer (model 214, Hamburg, Germany). BMI was used to classify the nutritional status as follows: BMI ≥than 95th percentile and overweight as a BMI ≥than percentile 85th among children of the same age and sex [46]. A Seca^®^ tape (model 201, Hamburg, Germany) was used to measure the waist circumference according to the previously described protocols [47].

### 2.3. Procedure

Research assistants attended the school during the 2019 school year in Chile, and carried out the assessments on those children who had the consent of parents and also gave their own assent. The evaluations were carried out over four separate sessions by a team of researchers trained in conducting the different tests. CRF was assessed in the first session: Prior to the testing sessions, the children performed a typical warm-up. In the second session, anthropometric assessments were carried out in a favourable space facilitated by the school. Then, lifestyle surveys were applied in the classrooms. A cognitive test was applied in a classroom and divided into the creativity plus memory (third session) and d2 test (final session). The questionnaires and cognitive instruments were completed individually and in the presence of researchers (they respected data confidentiality and clarified any potential doubts or questions). All the evaluations took place during the physical education classes in the morning. Parents completed the Pediatric Sleep Questionnaire during the first 2 weeks. Researchers returned to one more session to work with children whose measurements were missing. 

### 2.4. Statistical Analysis

Statistical analyses were performed using SPSS version 21.0 (SPSS Inc., Chicago, IL, USA). The Kolmogorov–Smirnov test and Levene’s test were used for the normal distribution of data and homogeneity of variances. Continuous variables were expressed as means and confidence intervals. Differences in the comparison between sex, CRF, and nutritional status groups were determined using an analysis of variance (ANOVA) test. The Bonferroni test was performed to detect differences between nutritional status groups.

To determine the association between creativity and memory with physical fitness, lifestyle, and anthropometrics parameters, a simple linear regression was used. The Chi-Squared test was applied to compare proportions according to sex and nutritional level. The multivariate analysis of variance (ANCOVA) was conducted with CRF groups and cognitive variables for grouping variables and sex, age and BMI as covariates. Cohen’s D was performed to determine the effect size. The significance level was set at *p* < 0.05.

## 3. Results

A total of 248 schoolchildren were included (Table 1). When analysing the sex groups separately, boys had significantly better scores in PA week (h) (2.58 vs. 2.16 *p* = 0.014) and $\dot{V}$O_2max_ (mL/kg/min) (42.18 vs. 40.62, *p* = 0.001) than girl peers. Girls performed significantly better in long-term memory than boys (8.96 vs. 8.02, *p* = 0.001). There were no significant differences in creativity (*p* = 0.699), concentration (*p* = 0.137), and selective attention (*p* = 0.246) according to sex.

When analysing the CRF (high/low) groups separately, schoolchildren with higher CRF reported significantly better long-term memory than lower CRF peers after adjusting for by age, sex, and BMI. There were no significant differences in creativity according to the CRF groups after adjusting for age, sex, and BMI. For selective attention and concentration, there were no significant differences, although schoolchildren with higher CRF obtained better scores than lower CRF peers (Table 2). 

When analysing the nutritional status groups separately, children with obesity reported lower creativity and long-term memory than normal weight children (*p* < 0.05). Additionally, children with obesity had lower scores in MD adherence (*p* < 0.05), $\dot{V}$O_2max_ (mL/kg/min) (*p* < 0.001) and presented more ST (*p* < 0.001) (Table 3).

In the total sample, creativity showed an association with $\dot{V}$O_2max_ (mL/kg/min) (β; 0.209, 95% CI; 0.02–0.40, *p* = *p* < 0.05) and with MD adherence (score) (β; 0.206, 95% CI; 0.01; 0.74, *p* = *p* < 0.05). Long-term memory showed an association with CRF (β; 1.076, 95% CI; 0.02–2.13, *p* = *p* < 0.05) and an inverse association with MD adherence (β; −0.155, 95% CI; −0.28–−0.03, *p* = *p* < 0.05) (Table 4). Moreover, long-term memory showed an inverse association with BMI (β; −0.167, 95% CI; −0.33–−0.01, *p* = *p* < 0.05).

## 4. Discussion

In the present study, the objective was to determine the association between creativity/memory and CRF, lifestyle parameters, and anthropometric measures in Chilean schoolchildren. A secondary aim was to compare creativity, memory, concentration, and selective attention according to CRF levels (i.e., high and low) and nutritional status. The main findings of this study were, first, creativity was associated with $\dot{V}$O_2max_ (mL/kg/min) and with MD adherence (score), while long-term memory was associated with CRF and BMI. Second, schoolchildren with higher CRF reported significantly better long-term memory than lower CRF peers after adjusting for age, sex, and BMI. Third, children with obesity reported lower creativity and long-term memory than normal weight children (*p* < 0.05). 

In this study, creativity was positively associated with $\dot{V}$O_2max_ and with MD adherence (score), this is in line with Latorre et al. [12], who reported that the highly creative group performed better in a CRF test than children with lower creativity levels. Moreover, the authors indicated that CRF was a predictor of creativity in schoolchildren. Likewise, Piya-amornphan et al. [48] reported a positive correlation between PA and creativity ability in adolescents. Similarly, Florence et al. [49] showed an association between diet quality and academic performance in Canadian schoolchildren, while Hidalgo et al. indicated that children with higher CRF had better creativity levels using the CREA test in Spanish adolescents [50]. Likewise, it has been reported that creativity is positively related to school achievement, and the authors demonstrated that middle school may be an especially creative period in adolescents’ development [51]. Therefore, it is essential to research how to stimulate creativity in school-age children [52]. We found that creativity reported a positive association with MD adherence. Concerning food habits, another study reported that a poor-quality diet negatively affects hippocampal function and thereby impairs performance of cognitive tasks such as creativity [53]. Similarly, a higher quality diet index and ideal diet (i.e., MD adherence score) were associated with better cognitive function in adolescents. The authors concluded that this association was strong enough to be relevant from a public health perspective [26]. Similarly, it has been indicated that diet habits are important among schoolchildren since they have high brain metabolic needs [24]. It is important to considerer that in Chilean schoolchildren, unhealthy lifestyle patterns such as low adherence to MD, could be explained by socio-economic status [54].

In this study, long-term memory was associated with CRF, and schoolchildren with higher CRF reported significantly better long-term memory after adjusting for age, sex, and BMI. Therefore, the study indicated that less fit preadolescents (i.e., lower CFR levels) had poorer memory recognition compared with more fit children [55]. Likewise, a cross-sectional study reported that CRF was positively correlated with working memory in male Spanish schoolchildren, and children in higher CRF and executive function categories obtained betters academic performance (i.e., mathematics and language) than children in lower categories [56]. Similarly, it has been reported that less fit preadolescent children had poorer relational memory task and smaller hippocampal volume compared to higher CRF preadolescent children, and the authors also indicated that it is fundamental to understand the neurocognitive benefits of an active lifestyle in educational and public health areas [57]. Another study showed that CRF was associated with working memory and reaction time [16]. Despite these associations, no relationships have been found between CRF and verbal working memory in primary schoolchildren [7]. Moreover, in this study, girls performed significantly better in long-term memory than boys. In this sense, a meta-analysis of sex differences in memory showed an overall female advantage in episodic memory task. Additionally, the authors indicated that the sex differences for verbal episodic memory tasks which are smaller in childhood than during other age periods, may indicate that fluctuating endogenous sex hormones and environmental influence contribute to the variation [58]. On the other hand, another study reported that several differences between gender in some executive function (i.e., reaction time in inhibition, cognitive flexibility) and language, were higher for girls but not in episodic memory [56].

In the present study, BMI was inversely associated with long-term memory. In addition, children with obesity reported lower creativity and long-term memory than normal weight children. In this sense, the evidence has shown an association between obesity and low cognition in children and adolescents, where children with obesity reported poor cognitive function compared with normal weight peers [59]. In this line, obesity in children has been associated with impaired cognitive function, poorer academic performance [60], reduction of executive cognitive performance on neuropsychological evaluations, and presented differences in brain structures related to learning, memory, and executive functions [61]. 

On the other hand, in this study, there were no significant differences in concentration and selective attention according to nutritional status groups. Contrary to our results, it has been reported that students with obesity presented worse results than normal peers in the d2 test (i.e., selective attention) [62]. In addition, previous studies indicated that deficits in attention, working memory, and sustained attention decrements do exist among overweight/obese adolescents [63,64]. However, other authors showed that there was no association between sustained attention and BMI [65]. In addition, another study reported that obese and non-obese children had similar results in attention and concentration [66]. Future studies on the current topic are therefore recommended to clarify these controversial results. Likewise, children with obesity had lower scores in MD adherence, $\dot{V}$O_2max_ (mL/kg/min), and presented more ST. In this sense, a previous study reported that obese students had lower scores in MD and $\dot{V}$O_2max_ compared with their normal weight peers [67].

For selective attention and concentration, there were no significant differences after adjusting for age, sex, and BMI, but schoolchildren with higher CRF obtained better scores than lower CRF peers. We believe that these results diverge from other studies since the sample was relatively small and the high CRF groups had more participants. In this sense, and contrary to our results, another study reported that children with high fitness levels (i.e., CRF, speed, and change of direction) had significantly better cognitive performance (i.e., selective attention and concentration using d2 tests) compared to children with low fitness levels. That study also indicated that CRF was the variable that explained the largest variance in the relationship between cognitive functioning and fitness [17]. Another previous study indicated that CRF was one of the variables of physical fitness that best explains the association with selective attention and concentration [68]. Reigal et al. [69] conducted a study of Spanish adolescents and showed that adolescents who practised more exercise per week obtained better scores in selective attention, concentration, and processing speed. CRF was also the best predictor of test scores to evaluate cognitive function. Pontifex et al. [70] have provided evidence that less fit children (i.e., low CRF) had reduced capacity to allocate attentional resources, greater response conflict, and slower processing speed than their more fit counterparts. Additionally, Ruíz-Ariza et al. [18] have indicated that variables relating to executive function such as memory, selective attention or concentration play important roles in cognitive performance among adolescents.

The main limitation of the present investigation is its cross-sectional design. These variables should be measured in a longitudinal study to clarify the direction of the associations. This study included the use of a convenience sample, and the results are not necessarily representative of the national population. Another limitation was that cognitive measures were obtained using a write-report instrument. Moreover, we did not carry out the analyses by biological maturation age or socioeconomic status. There is a limitation due to the Chilean educational system, in which children belonging to educational establishments with fewer resources most affected their healthy lifestyle (i.e., foods habits and PA patterns).

In conclusion, creativity was associated with CRF and MD adherence and memory was associated with CRF (i.e., CFR classification and $\dot{V}$O_2max_) and BMI in Chilean schoolchildren. Children with high CRF levels performed better in creativity and memory than those with low CRF levels. Likewise, children with obesity reported lower creativity and long-term memory than normal weight children. Likewise, children with obesity had lower scores in MD adherence, $\dot{V}$O_2max_ (mL/kg/min), and presented more ST.

Therefore, a physical fitness assessment and healthy lifestyle of schoolchildren should be essential for controlling the health of this population. In addition, an increase in CRF levels together with healthy food habits and normal BMI among these children should be a target for community- and school-based interventions to promote cognitive development such as creativity and memory.

## Figures and Tables

**Table 1:** Characteristics of the children participants according to sex groups at the level of anthropometry, cardiorespiratory fitness, physical activity patterns, creativity, memory, concentration, and selective attention.

	Total (248)	Girls (111)	Boys (137)	*p*-Value	Cohen’s d
Age (y)	11.70 (11.6,11.8)	11.58 (11.4, 11.8)	11.80 (11.6,12.0)	*p* = 0.132	0.193
**Anthropometric variables**					
BMI	22.06 (21.6, 22.5)	22.14 (21.5, 22.8)	21.99 (21.3, 22.7)	*p* = 0.746	−0.041
*Obesity prevalence*					
Normal weight, (*n* = /%)	109 (44)	46 (41.4)	63 (46)		0.383
Overweight, (*n* = /%)	76 (30.6)	39 (35.1)	37 (27)		
Obesity, (*n* = /%)	63 (25.4)	26 (23.4)	37 (27)		
WC (cm)	77.57 (76.3, 78.8)	76.70 (75.0, 78.4)	78.25 (76.4, 80.1)	*p* = 0.235	−0.015
**Lifestyle/fitness**					
Physical activity week (h)	2.39 (2.2, 2.6)	2.16 (1.9, 2.4)	2.58 (2.3, 2.8)	*p* = 0.014	0.154
Sleep duration (h/day)	8.48 (8.4, 8.6)	8.48 (8.3, 8.6)	8.48 (8.3, 8.6)	*p* = 0.977	0.003
ST (h/day)	2.90 (2.7, 3.1)	2.99 (2.7, 3.2)	2.82 (2.6, 3.0)	*p* = 0.337	−0.124
MD Adherence (score)	5.96 (5.63, 6.28)	5.85 (5.43, 6.37)	6.05 (5.63, 6.47)	*p* = 0.561	0.078
$\dot{V}$O_2max_ (mL/kg/min)	41.49 (41.0, 42.0)	40.62 (40.0, 41.3)	42.18 (41.5, 42.9)	*p* = 0.001	0.426
**Cognitive Measures**					
Creativity (score)	10.64 (10.2, 11.1)	10.54 (9.8, 11.2)	10.72 (10.1, 11.3)	*p* = 0.699	0.049
Memory (score)	8.45 (8.2, 8.7)	8.96 (8.5, 9.4)	8.02 (7.6, 8.4)	*p* = 0.001	−0.420
Concentration (score)	130.98 (126.7, 135.3)	127.36 (120.6, 134.1)	133.88 (128.3, 139.4)	*p* = 0.137	0.191
Selective Attention (score)	321.69 (311.4, 332.0)	314.91 (299.2, 330.6)	327.13 (313.4, 340.8)	*p* = 0.246	0.149

Data are presented as mean with 95% confidence interval (CI). Values of *p* < 0.05 were considered statistically significant. BMI: Body mass index; WC: Waist circumference; ST: Screen time; MD: Mediterranean diet; $\dot{V}$O_2max_: Maximal oxygen consumption. Obesity prevalence calculated based on the CDC criteria.

**Table 2:** Creativity, memory, concentration, and selective attention variables by cardiorespiratory fitness.

	High-CRF (164)	Low-CRF (69)	*p*-Value	Cohen’s d
Creativity (score)	11.15 (10.6, 11.7)	9.65 (8.8, 10.5)	NS	0.425
Memory (score)	8.88 (8.5, 9.2)	7.39 (6.9, 7.9)	*p* < 0.05	0.673
Concentration (score)	133.22 (127.7, 138.7)	126.43 (119.3, 133.6)	NS	0.199
Selective Attention (score)	324.09 (311.2, 337.0)	316.90 (299.9, 333.9)	NS	0.106

Data are presented as mean with 95% confidence interval (CI). Values of *p* < 0.05 were considered statistically significant after adjusting for sex, age, and BMI. CRF: cardiorespiratory fitness; NS: No considered statistically significant.

**Table 3:** Creativity, memory, concentration, selective attention variables, and lifestyle by nutritional status.

	Normal (109) A	Overweight (65) B	Obesity (74) C	*p*-Value	Post-Hoc
Creativity (score)	11.24 (10.6, 11.9)	11.19 (10.4, 12.0)	8.93 (8.1, 9.8)	*p* < 0.001	A > C
Memory (score)	9.33 (9.0, 9.7)	7.51(7.0, 8.0)	8.05 (7.4, 8.7)	*p* < 0.001	A > B,A > C
Concentration (score)	134.03 (127.9, 140.2)	132.72 (124.0, 141.4)	123.63 (115.5, 131.8)	0.138	
Selective Attention (score)	335.63 (320.6, 350.7)	307.69 (288.4, 327.0)	314.22 (293.5, 335.0)	0.053	
Screen time (h/day)	2.9 (2.69, 3.22)	2.5 (2.23, 2.78)	3.2 (2.92, 3.58)	*p* < 0.05	C > B
MD Adherence (score)	6.38 (5.93, 6.82)	6.36 (5.72, 7.0)	4.77 (4.17, 5.37)	*p* < 0.001	C < A,C < B
$\dot{V}$O_2max_ (mL/kg/min)	42.28 (41.48, 43.09)	41.63 (40.86, 42.40)	39.90 (39.11, 40.69)	*p* < 0.001	C < A,C < B

Data are presented as mean with 95% confidence interval (CI). Values of *p* < 0.05 were considered statistically significant.

**Table 4:** Association of creativity and memory score with socio-demographic, anthropometric, lifestyle, and cardiorespiratory fitness variables in schoolchildren.

	Creativity			Memory		
Outcomes	Beta	*p*-Value	Standardised Beta (SE)	Beta	*p*-Value	Standardised Beta (SE)
	(95% CI)			(95% CI)		
Age (y)	0.471 (0.02; 0.92)	*p* = 0.039	0.14 (0.23)	0.345 (0.05; 0.64)	*p* = 0.023	0.16 (0.15)
**Anthropometric variables**						
BMI (kg/m^2^)	−0.022 (−0.27; 0.22)	*p* = 0.859	−0.02 (0.12)	−0.167 (−0.33; −0.01)	*p* < 0.05	−0.27 (0.08)
WC (cm)	0.000 (−0.09; 0.09)	*p* = 0.994	0.00 (0.05)	0.004 (−0.06; 0.06)	*p* = 0.909	0.02 (0.03)
**Lifestyle**
PA/week (h)	0.347 (−0.04; 0.74)	*p* = 0.994	0.13 (0.20)	0.041 (−0.22; 0.30)	*p* = 0.753	0.02 (0.13)
PAC score	−0.025 (−0.10; 0.05)	*p* = 0.519	−0.05 (0.04)	0.005 (−0.05; 0.05)	*p* = 0.850	0.01 (0.03)
Sleep duration (h/day)	0.410 (−0.01; 0.94)	*p* = 0.132	0.10 (0.27)	−0.039 (−0.40; 0.32)	*p* = 0.832	−0.01 (0.18)
Screen time (h/day)	−0.293 (−0.66; 0.07)	*p* = 0.116	−0.11 (0.19)	0.020 (−0.22; 0.26)	*p* = 0.874	0.01 (0.12)
MD Adherence (score)	0.206 (0.01; 0.74)	*p* < 0.05	0.015 (0.99)	−0.155 (−0.28; −0.03)	*p* < 0.05	−0.17 (0.07)
**Physical fitness**						
$\dot{V}$O_2max_ (mL/kg/min)	0.209 (0.02; 0.40)	*p* < 0.05	0.22 (0.10)	−0.001 (−0.13; 0.12)	*p* = 0.988	0.00 (0.06)
High CRF (Ref. Low)	0.197 (−1.39; 1.79)	*p* = 0.807	0.03 (0.81)	1.076 (0.02; 2.13)	*p* < 0.05	0.21 (0.53)

Data shown represent beta and 95% confidence interval (95% CI), and standardized beta and standard error (SE). Values of *p* < 0.05 were considered statistically significant. Model adjusted by sex. BMI: Body mass index; WC: Waist circumference; PA: Physical activity; MD: Mediterranean diet; $\dot{V}$O_2max_: Maximal oxygen consumption; CRF: Cardiorespiratory fitness; PAC: Physical activity questionnaire.

## Data Availability

Not applicable.

## Abbreviations

BMI: Body mass index; CRF: Cardiorespiratory fitness; MD: Mediterranean diet; WC: Waist circumference; PA: Physical activity; ST: Screen time; O_2max_: Maximal oxygen consumption.
